# The Influence of Parent’s Cardiovascular Morbidity on Child Mental Health: Results from the National Health Interview Survey

**DOI:** 10.3390/children10010138

**Published:** 2023-01-11

**Authors:** Biplab Kumar Datta, Ashwini Tiwari, Elinita Pollard, Havilah Ravula

**Affiliations:** 1 Institute of Public and Preventive Health, Augusta University, Augusta, GA 30912, USA; 2 Department of Population Health Sciences, Medical College of Georgia, Augusta University, Augusta, GA 30912, USA; 3 Department of Psychological Sciences, Augusta University, Augusta, GA 30912, USA

**Keywords:** cardiovascular disease, child mental health, parents, chronic disease

## Abstract

Background: This study assessed the association between cardiovascular disease (CVD), the leading cause of death in the United States, among parents and child mental health. Methods: Our sample included 9076 children aged 6 to 17 years. Data were pooled from the 2016–2018 waves of the National Health Interview Survey. We fitted a logistic regression to obtain the odds ratios in favor of child mental health problems for parental CVD. We also fitted a multinomial logistic regression to obtain the odds in favor of the severity of mental health problems (i.e., minor, definite, and severe). Results: The adjusted odds of facing difficulties for a child of a parent with CVD were 1.64 (95% CI: 1.28–2.11) times that of their peers whose parents did not have CVD. The adjusted relative risk of facing minor and definite difficulties for a child of a parent with CVD were 1.48 (95% CI: 1.13–1.94) and 2.25 (95% CI: 1.47–3.46) times that of their peers of parents without CVD. Conclusions: The results suggest a strong association between child mental health and parental cardiovascular morbidity, demonstrating the need for the development or adaptation of existing public health interventions to facilitate mental health support for children of parents with CVD.

## 1. Introduction

Cardiovascular diseases (CVD) are the leading causes of morbidity and mortality worldwide [1]. In general, CVD risk is associated with prevalent risk factors such as childhood obesity, adulthood obesity, hypertension, consumption of low-quality foods, diabetes, and smoking [2,3,4,5]. Further, several studies offer evidence on independent links between CVD development and non-traditional predictors such as psychosocial stress, low socioeconomic status, negative affective reactions and emotions, sleep deprivation, and a lack of social support [6,7,8,9,10].

It is projected that by 2030, 40.5% of people in the United States will have some form of CVD [11]. Further, a growing concern has been the rising incidence of premature CVD, or CVD occurring among males aged ≤55 years and females aged ≤65 years [12], who are likely caregivers for children and adolescent populations. As such, it is critical to consider that the adverse effects of CVD may not only have implications for the individual with CVD, but downstream effects on child functioning and wellbeing.

To date, however, no studies have examined the direct or indirect impact of parental CVD on child health outcomes. Related literature on chronic pain, which may also be observed in CVD cases [13], could provide relevant insight on the plausible relationship between parental CVD and child mental health and behaviors. For example, recent evidence and a systematic review suggest that parental chronic pain and illness increase the risk of child psychological dysfunction, including increased internalizing symptoms for depression and anxiety [14,15]. Parental illness may also potentially affect child mental health through financial strain as well, which can increase parenting stress [16]. It is conceivable that in the case of CVD, reciprocal consequences of psychological distress may exacerbate CVD symptoms. In turn, these cyclical effects may extend the chronicity and severity of the disease, which are associated with exacerbating mental health symptoms among children [17]. As such, the investigation of the relationship between parental CVD conditions and children’s mental health may indeed be warranted.

CVD research in general is limited in its exploration of related household effects among children. Extant literature instead focuses on associations between CVD and factors known to increase the risk for poor child outcomes. For example, the risk of CVD morbidity and mortality among adults is linked to the development of chronic stress, depression, and anxiety [18,19,20]. While unexplored in the context of CVD, such poor parental mental health is a known risk factor for behavioral and mental health outcomes among children. Further, several empirical studies among younger and adolescent aged children of parents with mental health symptoms indicate that children may experience difficulties in social interactions and emotion regulation, as well as internalizing disorder symptoms such has depression and anxiety [21,22,23,24]. Yet, the potentially meaningful association between parental CVD and child outcomes has remained unexplored.

Addressing this gap is critical to help identify resources and relevant public health strategies to reduce the risk of mental health adversity among children. As such, the primary objective of the current study was to use nationally representative data to examine whether parental CVD diagnosis is associated with negative mental health among children under 18 years of age.

## 2. Materials and Methods

### 2.1. Data

We used data from the 2016, 2017, and 2018 waves of the National Health Interview Survey (NHIS). The NHIS data include a “Sample Child file” that provides information on children aged 17 and under; and a “Sample Adult file” that provides information on individuals aged 18 and over. Both files include unique identifiers for families within a household and persons within a family. Further, the “Sample Child file” includes information on a sample child’s relationship with a sample adult in the family. This allowed us to match children from the “Sample Child file” with their parents in the “Sample Adult file”. Our study sample included 9080 children aged 6 to 17 years, for whom we were able to match children and parents in both data files.

### 2.2. Measures

In the “Sample Child” survey, under the “Child Mental Health Brief Questionnaire” module, adult respondents were asked if the respondent thought that the child had difficulties in any of the following areas: (i) emotions; (ii) concentration; (iii) behavior; or (iv) being able to get along with other people. The response options were as follows: (i) no; (ii) yes—minor difficulties; (iii) yes—definite difficulties; and (iv) yes—severe difficulties. A child was deemed to have mental health problems if they reported having minor, definite, or severe difficulties in any of the aforementioned areas. We later checked sensitivity by defining mental health problems as having definite or severe difficulties. For the general analysis, the binary outcome variable took the value 1 if minor, definite, or severe difficulties were reported, and 0 if otherwise. For the sensitivity analysis, the binary outcome variable took the value 1 if definite or severe difficulties were reported, and 0 if otherwise.

As a robustness check, we considered four mental health indicators as follows: (i) the child has many worries or often seemed worried; (ii) the child is often unhappy, depressed, or tearful; (iii) the child is generally well behaved and usually does what adults request; and (iv) the child has good attention span. Adult respondents were asked whether in the past six months preceding the survey, these conditions were not, somewhat, or certainly true for the child. A child was deemed to have the condition if it was reported somewhat or certainly true.

In the “Sample Adult” module, respondents were asked if they were ever told by a doctor or other health professionals that they had (i) coronary heart disease; (ii) angina pectoris; (iii) myocardial infarction; and (iv) any other heart condition or heart disease. A respondent was determined to have cardiovascular morbidity if they reported having any of these conditions.

### 2.3. Statistical Analysis

We first assessed the frequency of children having no, minor, definite, and severe problems by parent’s CVD condition. We performed adjusted Wald tests to examine whether the differences were statistically significant. Level of significance was set at 5% level.

Next, we estimated a binomial logistic regression to obtain the odds ratios in favor of mental health problem for parent’s CVD condition indicator. Our outcome variable was a binary variable indicating whether the child had a mental health problem or not. Our explanatory variable was another binary variable indicating whether the parent of the child had CVD or not.

We then estimated a multivariable specification where we controlled for several sociodemographic characteristics of children and their parents. Correlates related to children include age, sex, and race. Correlates related to parents include age, sex, marital status, educational attainment, employment status, and self-reported health conditions. We also accounted for household income (as share of federal poverty level threshold) and U.S. Census Bureau region fixed effects. Of note, these covariates were not included in the model to assess their relationship with the outcome variable, but to examine whether the relationship between child mental health problems and parent’s CVD condition persisted after taking these sociodemographic attributes into account.

As a sensitivity check, both univariate and multivariable specifications were also estimated for child mental health problems, defined as having “definite or severe” difficulties instead of “minor, definite, or severe” difficulties. Next, separate logistic regressions were estimated for the four mental health conditions to check the robustness of the original results. The outcome variable for each condition was a binary variable indicating whether that condition was true for the child.

Lastly, we estimated a multinomial logistic regression to assess the degree of severity of the problem. We obtained unadjusted and adjusted relative risk ratios in favor of having (i) minor difficulties; (ii) definite difficulties; and (iii) severe difficulties, relative to the base outcome of “no difficulty” for the parent’s CVD indicator. All estimates were obtained using complex survey weight of the NHIS and using Stata 17.0 software.

## 3. Results

Around 6% of the children in our sample had parents with CVD. Table 1 presents the descriptive statistics of the study participants by parent’s CVD condition. For the majority of the children (~70%), data on the mother’s information was included. Approximately 60% of parents were married in child households. While the children’s and their parents’ sex and race, in general, were comparable across the exposure (i.e., parent had CVD) and the control (i.e., parent did not have CVD) groups, the percent of parents with marital dissolution/disruption (i.e., widowhood, divorce, or separation) was higher (29% vs. 19%) among those who had CVD. Parents with CVD had lower (66% vs. 74%) employment and poorer self-reported health (Table 1).

Among children whose parents did not have CVD, 77.0% reported no difficulties in in emotions, concentration, behavior, or being able to get along with other people. This proportion was 15.0 percentage-points (pp) lower among children whose parents had CVD. Though the difference between children with parents having and not having CVD was not statistically significant for the “severe” difficulties outcome, statistically significant differences were observed for both “minor” and “definite” difficulties outcomes (Table 2).

Table 3 presents the crude and adjusted odds of having mental health problems. The odds of having minor, definite, or severe difficulties for children whose parents had CVD were 2.1 times that of their counterparts whose parents did not have CVD. When the socioeconomic and demographic characteristics, along with parent’s self-reported health status, were accounted for, the adjusted odds ratio slightly decreased to 1.6. Similar were the results for the odds of having definite, or severe difficulties. Children whose parents had CVD were 2.5 times more likely to have definite, or severe difficulties compared with children whose parents did not have CVD. Like the original specification, the adjusted odds ratio in the sensitivity analysis was also slightly smaller.

Results of the robustness analyses for different mental health indicators are presented in Table 4. Compared with children whose parents did not have CVD, children whose parents had CVD were 1.3 times more likely to have many worries or often seemed worried. Children whose parents had CVD were also more likely to often be unhappy, depressed, or tearful. While no statistically significant difference in being well behaved or usually doing what adults request was observed between children with parents having and not-having CVD, children whose parents had CVD were 29.3% less likely to have a good attention span.

Lastly, the results of the multinomial specifications are presented in Table 5. Relative to the base outcome of no difficulty, children whose parents had CVD were 1.8, 3.1, and 2.3 times more likely to have minor-, definite-, and severe- difficulties, respectively, than those of their peers whose parents did not have CVD. The adjusted relative risk ratios were similar, though not statistically significant for the severe difficulties outcome. 

## 4. Discussion

To the best of our knowledge, this is the first study to investigate associations between parental CVD with child mental health outcomes. Our findings indicate that children of parents with CVD may be more vulnerable to experiencing poor mental health outcomes compared to children whose parents do not have CVD. The findings of the current study are in concordance with the studies illustrating that parental illness may increase risk of mental health difficulties and psychosocial maladjustment among children and adolescents [25,26]. These results are also be in line with previous research that suggests parental illness can impact children’s health-related quality of life, psychosomatic complaints, and life satisfaction [27,28,29].

Findings from the current study may be explained by several mechanisms. For example, some studies indicate that parental physical illness may increase existing, or create new caring demands placed on a child in a household [30,31]. For example, young caregivers under 18 years of age, referred to as ‘young carers’ [32], may be responsible for domestic, social, and emotional support responsibilities. The role-reversal and potential disruptions to routine functioning, may place them at risk of ‘young carer penalty’ [33], a term encompassing the new strains associated with caregiving across a youth’s personal and external ecological systems. Drawing from evidence on youth carers, higher levels of caregiving among the youth of parents with chronic illness is associated with an increased likelihood of mental illness [34,35] and potential isolation [36]. However, we caution that more research is needed to understand the role of caregiving responsibilities among the youth of parents with CVD in predicting youth well-being.

Another explanation may be the effects of CVD on parent–child interactions. Although there is a dearth of literature in this area, and while beyond the scope of this paper, it is possible that the relationship between parent and child is negatively affected by the development or long-term burden of CVD in the household. As illustrated by McDowell and Parke’s [37] tripartite model, parent–child interactions can have a significant influence over a child’s development, including social competence. Relatedly, negative peer interactions, such as ostracization, are linked to poor mental health outcomes among children [38]. Taken together, child mental health outcomes among parents with CVD may be an indirect byproduct of a confluence of factors.

Notably, while this study could not examine parental mental health relative to CVD diagnosis or the temporality of these variables, pre-existing mental illness among parents may have a proximal effect on child mental health, as a family history of mental disorders, such as depression, is considered to be a strong predictor of developing disorders in adolescence [39]. It is also of importance to consider the role of other aforementioned psychosocial factors linked to CVD prognosis among adults. Recent findings suggest that perceptions of poor social support and a higher burden of stress among parents are likely to negatively impact parenting skills and child wellbeing [40]. The effects of psychosocial domains of influence on child health should be of rising interest to researchers studying at-risk parents for CVD following the COVID-19 pandemic, which heightened social isolation and mental illness for populations worldwide.

Our findings should be interpreted with some caution. First, in these cross-sectional data, we were not able to identify whether a parent’s CV condition preceded a child’s mental health condition and vice versa. As such, we cannot assume a causal interpretation. Second, due to the survey design, we were limited to data coming from one parent. Third, the CV conditions were self-reported; there was no information on the severity or frequency of the condition. Despite these limitations, our analyses using nationally representative data present a strong association between parental CVD condition and children’s mental health outcomes.

## 5. Conclusions

This study provides an important contribution in understanding the association between parental cardiovascular morbidity and child mental health. Our findings suggest that children of parents with CVD may experience significant mental health adversity. While more longitudinal research is needed to understand how parental CVD relates to child mental illness, our findings suggest that children should be considered as an important target in therapeutic interventions for parents with CVD to prevent downstream adverse outcomes.

Of importance to note, the burden of CVD in the United States is substantial in magnitude and there are sizable disparities by socioeconomic status [41]. Children from lower SES groups may have a disproportionately higher risk of having mental health issues, channeled through their parents’ cardiovascular health. This concern is particularly relevant to the pandemic, which has exacerbated the financial constraint across many households [42], and has had prognostic CVD implications for patients [43]. With an ever-growing population of adults who may be living with cardiovascular morbidity, future directions must recognize that child outcomes may be influenced by parental health. The promotion of existing cardiovascular prevention strategies may therefore not only have implications for improving CVD incidence, but for potentially preventing long-term child mental health illness.

## Figures and Tables

**Table 1:** Descriptive statistics.

	Percentage of Children		
	All	Parent Did Not Have CVD	Parent Had CVD
Child’s sex			
Male	51.21	51.33	49.35
Female	48.79	48.67	50.65
Race			
White	52.48	52.16	57.41
Black	14.27	14.31	13.76
Hispanic	23.89	24.2	19.18
Other	9.36	9.34	9.66
Parent’s sex			
Male	29.43	29.26	31.98
Female	70.57	70.74	68.02
Parent’s marital status			
Married	61.27	61.54	57.25
Never married	12.8	13.01	9.55
Living with partner	6.35	6.48	4.27
Widowed/divorced/separated	19.58	18.97	28.93
Parent’s education			
College graduate	36.26	36.29	35.83
<High school diploma	12.85	13	10.59
High school graduate	19.01	19.21	16.03
Some college	31.87	31.5	37.56
Parent’s employment			
Not employed	26.27	25.75	34.26
Employed	73.73	74.25	65.74
Parent’s self-reported health			
Excellent	30.6	31.63	14.86
Very good	34.62	35.22	25.41
Good	25.74	25.25	33.14
Fair	7.43	6.67	19.07
Poor	1.62	1.23	7.51
Household income			
≥400% of FPL	30.39	30.53	28.29
<100% of FPL	19.8	19.48	24.67
100% to <200% of FPL	22.8	22.96	20.35
200% to <400% of FPL	27.01	27.03	26.69
Observations	9076	8513	563

Note: Estimates were obtained using complex survey weights. Parent’s marital status was not available for 5 observations. Parent’s education was not available for 13 observations. Parent’s employment status was not available for 4 observations. Parent’s self-reported health status was not available for 1 observation. Household income was not available for 399 observations.

**Table 2:** Share of children having difficulties in emotions, concentration, behavior, or being able to get along with other people—by parent’s cardiovascular diseases.

	No Difficulty	Difficulties		
		Minor	Definite	Severe
Parent’s CVD				
No	77.03	16.88	4.61	1.48
	(75.89, 78.16)	(15.89, 17.87)	(4.05, 5.16)	(1.18, 1.79)
Yes	62.00	23.89	11.39	2.73
	(56.92, 67.07)	(19.58, 28.19)	(8.02, 14.77)	(1.27, 4.19)
Difference				
CVD—No CVD	−15.03 ***	7.00 **	6.79 ***	1.24
	(−20.22, −9.85)	(2.6, 11.41)	(3.37, 10.21)	(−0.24, 2.72)
Observations	6832	1608	477	159

Note: Estimates were obtained using complex survey weights. *** *p* < 0.001, ** *p* < 0.01.

**Table 3:** Crude and adjusted odds ratios in favor of difficulties in emotions, concentration, behavior, or being able to get along with other people.

	Original Specification		Sensitivity Analysis	
	Crude Odds Ratio	Adjusted Odds Ratio	Crude Odds Ratio	Adjusted Odds Ratio
CVD	2.056 ***	1.641 ***	2.535 ***	1.862 ***
	(1.643, 2.571)	(1.279, 2.105)	(1.843, 3.487)	(1.295, 2.677)
Child’s age	1.016	1.014	1.028	1.030
	(0.998, 1.033)	(0.993, 1.036)	(0.999, 1.059)	(0.997, 1.065)
Child’s sex				
Male	Ref.	Ref.	Ref.	Ref.
Female	0.627 ***	0.609 ***	0.517 ***	0.509 ***
	(0.561, 0.701)	(0.541, 0.685)	(0.424, 0.630)	(0.412, 0.630)
Race				
White	Ref.	Ref.	Ref.	Ref.
Black	0.988	0.710 **	0.859	0.542 **
	(0.816, 1.196)	(0.565, 0.892)	(0.642, 1.151)	(0.369, 0.798)
Hispanic	0.698 ***	0.553 ***	0.724 *	0.493 ***
	(0.596, 0.816)	(0.457, 0.669)	(0.561, 0.934)	(0.364, 0.667)
Other	0.816 *	0.758 *	0.778	0.645 *
	(0.666, 0.999)	(0.611, 0.941)	(0.547, 1.107)	(0.447, 0.933)
Parent’s age	0.992	0.992	0.989	0.988
	(0.984, 1.000)	(0.982, 1.002)	(0.975, 1.003)	(0.972, 1.005)
Parent’s sex				
Male	Ref.	Ref.	Ref.	Ref.
Female	1.593 ***	1.352 ***	2.031 ***	1.634 ***
	(1.374, 1.846)	(1.147, 1.595)	(1.549, 2.662)	(1.222, 2.186)
Parent’s marital status				
Married	Ref.	Ref.	Ref.	Ref.
Never married	1.748 ***	1.550 ***	1.931 ***	1.438
	(1.466, 2.085)	(1.233, 1.949)	(1.465, 2.545)	(0.999, 2.070)
Living with partner	1.796 ***	1.658 ***	2.655 ***	2.352 ***
	(1.418, 2.274)	(1.283, 2.142)	(1.867, 3.776)	(1.624, 3.406)
Widowed/divorced/separated	1.709 ***	1.471 ***	2.277 ***	1.676 ***
	(1.477, 1.978)	(1.236, 1.750)	(1.796, 2.887)	(1.262, 2.226)
Parent’s education				
College graduate	Ref.	Ref.	Ref.	Ref.
< High school diploma	1.211	0.957	1.374	0.755
	(0.983, 1.491)	(0.733, 1.248)	(0.960, 1.965)	(0.492, 1.156)
High school graduate	1.182	0.903	1.446 *	0.835
	(0.999, 1.398)	(0.732, 1.113)	(1.077, 1.941)	(0.598, 1.168)
Some college	1.448 ***	1.086	1.373 *	0.774
	(1.254, 1.673)	(0.914, 1.292)	(1.073, 1.756)	(0.594, 1.009)
Parent’s employment				
Not employed	Ref.	Ref.	Ref.	Ref.
Employed	0.676 ***	0.812 **	0.672 ***	0.996
	(0.598, 0.766)	(0.694, 0.951)	(0.548, 0.825)	(0.785, 1.263)
Parent’s self-reported health				
Excellent	Ref.	Ref.	Ref.	Ref.
Very good	1.656 ***	1.596 ***	1.812 ***	1.685 **
	(1.391, 1.971)	(1.326, 1.921)	(1.297, 2.530)	(1.190, 2.385)
Good	2.526 ***	2.455 ***	3.376 ***	3.137 ***
	(2.114, 3.017)	(2.017, 2.988)	(2.423, 4.702)	(2.192, 4.490)
Fair	3.679 ***	3.305 ***	5.480 ***	4.694 ***
	(2.913, 4.646)	(2.547, 4.288)	(3.798, 7.908)	(3.109, 7.086)
Poor	7.088 ***	4.856 ***	11.865 ***	7.873 ***
	(4.702, 10.686)	(3.128, 7.539)	(7.085, 19.871)	(4.462, 13.891)
Household income				
≥400% of FPL	Ref.	Ref.	Ref.	Ref.
<100% of FPL	1.752 ***	1.073	2.416 ***	1.570 *
	(1.474, 2.082)	(0.832, 1.384)	(1.802, 3.241)	(1.031, 2.392)
100% to <200% of FPL	1.384 ***	1.037	1.979 ***	1.516 *
	(1.172, 1.635)	(0.837, 1.284)	(1.499, 2.613)	(1.094, 2.100)
200% to <400% of FPL	1.176	0.970	1.073	0.886
	(0.997, 1.386)	(0.802, 1.172)	(0.789, 1.459)	(0.628, 1.249)
Observations	9076	8663	9076	8663

Note: Estimates were obtained using complex survey weights. *** *p* < 0.001, ** *p* < 0.01, * *p* < 0.05. The multivariable specification controls for U.S. region fixed effects. In original specification, having mental health problem was defined as having minor-, definite-, or severe difficulties. In the sensitivity analysis, having mental health problem was defined as having definite, or severe difficulties.

**Table 4:** Crude and adjusted odds ratios in favor of mental health indicators.

	Has Many Worries/Often Seemed Worried	Often Unhappy/ Depressed/ Tearful	Well Behaved/ Usually Does What Adults Request	Has Good Attention Span
A. Unadjusted				
CVD	1.754 ***	1.866 ***	0.617	0.604 **
	(1.406, 2.187)	(1.440, 2.419)	(0.349, 1.090)	(0.447, 0.818)
Observations	9070	9070	9075	9072
B. Adjusted				
CVD	1.307 *	1.401 *	0.764	0.717 *
	(1.034, 1.653)	(1.060, 1.852)	(0.414, 1.411)	(0.516, 0.996)
Observations	8658	8660	8663	8660

Note: Estimates were obtained using complex survey weights. *** *p* < 0.001, ** *p* < 0.01, * *p* < 0.05. The multivariable specification controls for child’s age, sex, and race; parent’s age, sex, marital status, educational attainment, employment status, and self-reported health status; household income and U.S. region fixed effects.

**Table 5:** Relative risk ratios in favor of difficulties in emotions, concentration, behavior, or being able to get along with other people.

	Base Outcome: No Difficulty	Outcome I: Minor Difficulties	Outcome II: Definite Difficulties	Outcome III: Severe Difficulties
A. Unadjusted				
CVD	Ref.	1.758 ***	3.073 ***	2.282 **
		(1.363, 2.268)	(2.128, 4.439)	(1.265, 4.118)
B. Adjusted				
CVD	Ref.	1.480 **	2.250 ***	1.605
		(1.129, 1.938)	(1.465, 3.455)	(0.890, 2.897)

Note: Estimates were obtained using complex survey weights. *** *p* < 0.001, ** *p* < 0.01. The multivariable specification adjusted for the following covariates: child’s age, child’s sex, child’s race, parent’s age, parent’s sex, parent’s marital status, parent’s educational attainment, parent’s employment status, parent’s self-reported health, household income as ratio of Federal Poverty Level, and U.S. region fixed effects.

## Data Availability

We used Public Use version of the NHIS, which is publicly available to download from the National Center for Health Statistics, Centers for Disease Control and Prevention, by accessing the following URL: https://www.cdc.gov/nchs/nhis/data-questionnaires-documentation.htm, accessed on 5 January 2023.
